# Two types of fatigue in cancer patients

**DOI:** 10.1038/bjc.2011.528

**Published:** 2012-01-03

**Authors:** A Giacalone, M Spina, M Berretta, U Tirelli

**Affiliations:** 1 Department of Medical Oncology, National Cancer Institute, Via Franco Gallini 2, 33081 Aviano (PN), Italy

**Sir,**

The very interesting data presented by Singer *et al* (2011), on prevalence of fatigue at three time points, that is, the beginning of inpatient treatment, at discharge and half a year after diagnosis, raise some questions and comments. First of all, it is not reported when fatigue present at the beginning of inpatient treatment started; that is, fatigue was present weeks, months or years before the diagnosis of cancer. In other words was fatigue related to the presence of cancer or was it antecedent, and not related to cancer presence? In fact, it could be that some patients had a chronic fatigue present many months or years before the diagnosis of cancer; and therefore, fatigue could not be due to cancer, but was already present for a different reason, for example, a chronic fatigue syndrome (CFS). Moreover, it would be very interesting to know how many of the patients with fatigue half a year after diagnosis were fatigued years after, obviously in case they were alive. These questions are based on the fact that in our opinion there are two types of fatigue in cancer patients needing different symptom monitoring and management: one that could be defined as cancer-related fatigue and is that nicely described by Singer and co-workers, and the other could be defined as CFS-cancer related and it is observed before diagnosis and after therapy, in both cases not obviously related to cancer.

In fact, to better understand the different course of fatigue in cancer patients and in survivors, we need different definitions.

Cancer-related fatigue, necessarily associated with cancer and its treatments, has to be adopted in patients with active disease, on treatments, and until 1 year from diagnosis. In this population, there are different causes for fatigue, including anaemia, endocrine and metabolic disorders, cardiovascular and renal dysfunctions, and this is the condition nicely reported by Singer and colleagues.

Instead, the chronic and persistent fatigue experienced before the diagnosis or by long-term cancer survivors is not necessary related to or caused by cancer and its treatments. These patients show a particular type of fatigue similarly to the fatigue experienced by patients affected by CFS, which accordingly to the CDC case definition (Fukuda *et al*, 1994) is a severe fatigue usually associated with impairment in short-term memory or concentration (severe enough to cause substantial reduction in previous levels of occupation, educational source or personal activities); post-exertional malaise lasting >24 h; soar throat; tender cervical or axillary lymph nodes; muscle pain; multi-joint pain without joint swelling or redness; headaches of a new type for pattern or severity; and unrefreshed sleep.

As we have previously suggested (Simonelli *et al*, 2008), we believe that, in particular in long-term cancer survivors, fatigue can be a chronic/persistent condition associated with disrupting symptoms similar to CFS (Fukuda *et al*, 1994). For these reasons, we believe that fatigue in these conditions may be considered a subtype of CFS and could be defined as CFS-cancer related. Further studies could be carried out in these settings to better define these two entities, that is, cancer-related fatigue and CFS-cancer related.
